# Validation of a real-time PCR panel for detection and quantification of nine pathogens commonly associated with canine infectious respiratory disease

**DOI:** 10.1016/j.mex.2023.102476

**Published:** 2023-11-11

**Authors:** Junsheng Dong, Wai Ning Tiffany Tsui, Xue Leng, Jinping Fu, Molly Lohman, Joseph Anderson, Vaughn Hamill, Nanyan Lu, Elizabeth Poulsen Porter, Mark Gray, Tesfaalem Sebhatu, Susan Brown, Roman Pogranichniy, Heng Wang, Lance Noll, Jianfa Bai

**Affiliations:** a Kansas State Veterinary Diagnostic Laboratory, Kansas State University, Manhattan, KS 66506, United States; b Department of Diagnostic Medicine/Pathobiology, Kansas State University, Manhattan, KS, United States; c Yangzhou University College of Veterinary Medicine, Yangzhou, Jiangsu, China; d Jilin Agricultural University, Changchun, Jilin, China; e Division of Biology, Kansas State University, Manhattan, Kansas, United States

**Keywords:** Validation method for a canine respiratory PCR assay, Canine infectious respiratory disease, CIRD, PCR assay, Diagnostic validation

## Abstract

Canine infectious respiratory disease (CIRD) is a complicated respiratory syndrome in dogs [1], [2], [3]. A panel PCR was developed [4] to detect nine pathogens commonly associated with CIRD: *Mycoplasma cynos, Mycoplasma canis, Bordetella bronchiseptica;* canine adenovirus type 2, canine herpesvirus 1, canine parainfluenza virus, canine distemper virus, canine influenza virus and canine respiratory coronavirus [5], [6], [7], [8], [9], [10], [11], [12], [13], [14], [15], [16]. To evaluate diagnostic performance of the assay, 740 nasal swab and lung tissue samples were collected and tested with the new assay, and compared to an older version of the assay detecting the same pathogens except that it does not differentiate the two *Mycoplasma* species. Results indicated that the new assay had the same level of specificity, but with higher diagnostic sensitivity and had identified additional samples with potential co-infections. To confirm the new assay is detecting the correct pathogens, samples with discrepant results between the two assays were sequence-confirmed. Spiking a high concertation target to samples carrying lower concentrations of other targets was carried out and the results demonstrated that there was no apparent interference among targets in the same PCR reaction. Another spike-in experiment was used to determine detection sensitivity between nasal swab and lung tissue samples, and similar results were obtained.

• A nine-pathogen CIRD PCR panel assay had identified 139 positives from 740 clinical samples with 60 co-infections;
• High-concentration target does not have apparent effect on detecting low-concentration targets;
• Detection sensitivity were similar between nasal swab and lung tissue samples.

## Graphical abstract

**Figure:**
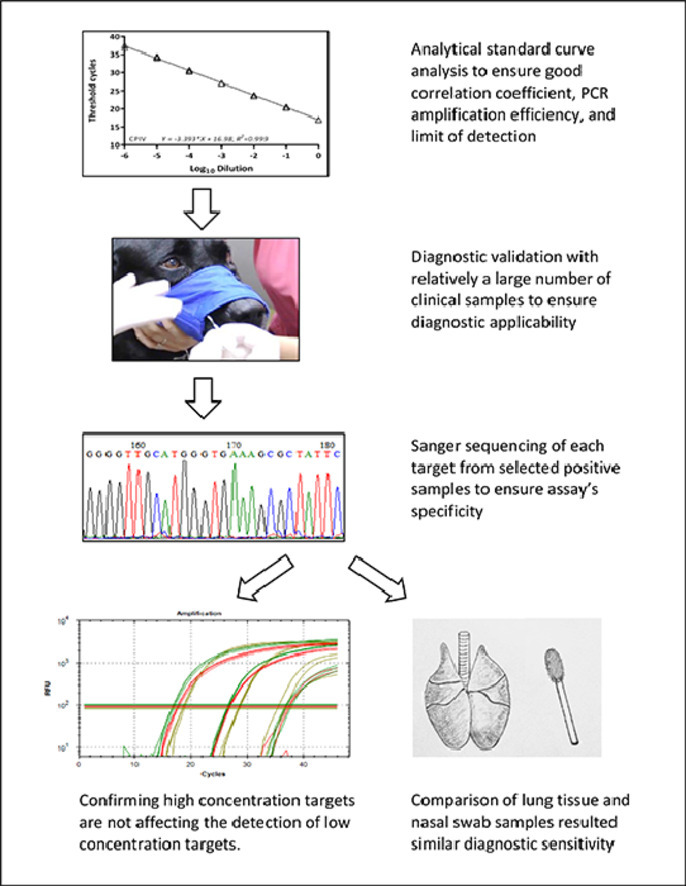

Specifications table

**Table:** 

Subject area:	Veterinary Science and Veterinary Medicine
More specific subject area:	Molecular detection of animal infectious disease
Name of your method:	Validation method for a canine respiratory PCR assay
Name and reference of original method:	Development of a three-panel multiplex real-time PCR assay for simultaneous detection of nine canine respiratory pathogens. J Microbiol Methods. 2022 Jun 23:106,528. https://doi.org/10.1016/j.mimet.2022.106528.
Resource availability:	https://www.ncbi.nlm.nih.gov/ https://blast.ncbi.nlm.nih.gov/Blast.cgi https://www.qiagen.com/us https://bioinfo.ut.ee/primer3-0.4.0/ https://bioedit.software.informer.com/7.2/

## Method details

### Assay layout

Nine major canine respiratory pathogens [5], [6], [7], [8], [9], [10], [11], [12], [13], [14], [15], [16] were included in the panel assay with three PCR reaction panels [4]. Panel 1 includes *Mycoplasma cynos (M. cynos), Mycoplasma canis (M. canis)*, and *Bordetella bronchiseptica (B. br);* Panel 2 has canine adenovirus type 2 (CAdV-2), canine herpesvirus 1 (CHV-1), and canine parainfluenza virus (CPIV); and Panel 3 detects canine distemper virus (CDV), canine influenza virus (CIA), and canine respiratory coronavirus (CRCoV). A canine housekeeping gene, Glyceraldehyde 3-Phosphate Dehydrogenase (cGAPDH), is used as an internal control, and is included in Panel 3 to monitor nucleic acid extractions and potential PCR inhibitions.

### Primer and probe design

Detailed information for the primers and probes was provided in Table 1 of Dong et al., 2022 [4]. Briefly, all available target sequences of the nine canine respiratory pathogens and the cGAPDH gene were downloaded from the GenBank database (https://www.ncbi.nlm.nih.gov/), and aligned in CLC Main Workbench 20 (Qiagen, Valencia, CA, USA). Conserved regions were used for primer and probe designs (Primer 3.0, https://bioinfo.ut.ee/primer3-0.4.0/). Melting temperature (Tm) of approximately 60 °C were deliberately designed for all real-time PCR primers, and 63 °C and 65 °C for all probes to allow for concurrent testing of each subpanel reactions on the same machine run.

**Table 1:** Real-time PCR validation data of 139 positive samples for each of the nine pathogens included in this canine respiratory PCR panel assay.

Sample ID	Panel 1			Panel 2			Panel 3			No. of positive
	M. cynos	M. canis	B. br	CAdV2	CHV1	CPIV	CDV	CIV	CrCoV	
1	30.35	22.65	0.00	0.00	20.97	0.00	0.00	0.00	0.00	**3**
2	14.49	21.24	0.00	0.00	0.00	0.00	0.00	0.00	0.00	**2**
3	0.00	0.00	0.00	0.00	0.00	0.00	0.00	0.00	35.84	**1**
4	0.00	0.00	0.00	26.22	0.00	34.63	0.00	0.00	0.00	**2**
5	26.66	0.00	0.00	0.00	0.00	36.86	0.00	0.00	0.00	**2**
6	0.00	0.00	36.06	0.00	0.00	0.00	0.00	0.00	0.00	**1**
7	21.15	0.00	0.00	0.00	0.00	0.00	0.00	0.00	0.00	**1**
8	0.00	21.16	0.00	0.00	0.00	0.00	0.00	0.00	0.00	**1**
9	0.00	25.80	0.00	0.00	0.00	0.00	0.00	0.00	0.00	**1**
10	0.00	15.82	0.00	0.00	0.00	0.00	36.86	0.00	0.00	**2**
11	0.00	30.01	0.00	0.00	0.00	0.00	0.00	0.00	0.00	**1**
12	0.00	26.02	0.00	0.00	0.00	0.00	0.00	0.00	0.00	**1**
13	33.19	23.25	0.00	0.00	0.00	0.00	0.00	0.00	0.00	**2**
14	23.98	20.86	0.00	0.00	0.00	0.00	0.00	0.00	0.00	**2**
15	0.00	0.00	0.00	0.00	0.00	0.00	33.21	0.00	0.00	**1**
16	0.00	17.24	0.00	0.00	0.00	0.00	0.00	0.00	0.00	**1**
17	0.00	20.80	0.00	0.00	0.00	0.00	35.71	0.00	0.00	**2**
18	27.97	0.00	0.00	0.00	0.00	0.00	34.65	0.00	0.00	**2**
19	0.00	0.00	0.00	0.00	0.00	0.00	35.05	0.00	0.00	**1**
20	20.10	23.23	0.00	0.00	0.00	0.00	0.00	0.00	0.00	**2**
21	0.00	21.63	0.00	0.00	0.00	0.00	36.80	0.00	0.00	**2**
22	0.00	0.00	0.00	0.00	0.00	0.00	32.06	0.00	0.00	**1**
23	0.00	0.00	0.00	0.00	0.00	0.00	34.07	0.00	0.00	**1**
24	12.56	0.00	0.00	0.00	0.00	0.00	29.59	0.00	0.00	**2**
25	12.67	0.00	0.00	0.00	0.00	0.00	23.15	0.00	0.00	**2**
26	27.83	0.00	0.00	0.00	0.00	0.00	21.95	0.00	0.00	**2**
27	0.00	0.00	0.00	0.00	0.00	0.00	33.20	0.00	0.00	**1**
28	0.00	0.00	0.00	0.00	0.00	0.00	32.90	0.00	0.00	**1**
29	0.00	0.00	0.00	0.00	0.00	0.00	34.15	0.00	0.00	**1**
30	0.00	19.70	0.00	0.00	0.00	0.00	31.52	0.00	0.00	**2**
31	0.00	0.00	0.00	0.00	0.00	0.00	32.54	0.00	0.00	**1**
32	0.00	0.00	0.00	0.00	0.00	0.00	36.38	0.00	0.00	**1**
33	0.00	15.40	0.00	0.00	0.00	0.00	0.00	0.00	0.00	**1**
34	0.00	29.49	0.00	0.00	0.00	0.00	0.00	0.00	0.00	**1**
35	0.00	20.70	0.00	0.00	0.00	0.00	0.00	0.00	0.00	**1**
36	0.00	0.00	0.00	0.00	0.00	0.00	36.54	0.00	0.00	**1**
37	21.04	0.00	0.00	0.00	0.00	0.00	0.00	0.00	0.00	**1**
38	25.68	17.58	0.00	0.00	0.00	0.00	0.00	0.00	0.00	**2**
39	30.14	0.00	0.00	0.00	0.00	0.00	0.00	0.00	0.00	**1**
40	0.00	0.00	0.00	0.00	20.74	22.55	0.00	0.00	0.00	**2**
41	14.97	20.27	0.00	0.00	0.00	32.09	0.00	0.00	0.00	**3**
42	18.70	0.00	0.00	0.00	0.00	0.00	0.00	0.00	0.00	**1**
43	22.98	0.00	0.00	0.00	0.00	0.00	0.00	0.00	0.00	**1**
44	34.16	0.00	0.00	0.00	0.00	0.00	0.00	0.00	0.00	**1**
45	23.32	21.37	0.00	0.00	0.00	0.00	0.00	0.00	0.00	**2**
46	16.91	14.01	0.00	0.00	0.00	0.00	0.00	0.00	29.85	**3**
47	0.00	0.00	0.00	0.00	0.00	0.00	19.26	0.00	0.00	**1**
48	26.11	0.00	0.00	0.00	0.00	0.00	0.00	0.00	0.00	**1**
49	0.00	24.20	25.12	35.38	0.00	0.00	21.74	0.00	0.00	**4**
50	0.00	0.00	0.00	0.00	0.00	0.00	25.71	0.00	0.00	**1**
51	0.00	0.00	0.00	0.00	0.00	0.00	22.25	0.00	0.00	**1**
52	0.00	23.73	24.30	0.00	0.00	26.47	21.48	0.00	33.47	**5**
53	0.00	20.50	0.00	0.00	0.00	27.60	0.00	0.00	0.00	**2**
54	0.00	0.00	18.02	10.50	0.00	29.20	0.00	0.00	0.00	**3**
55	22.59	0.00	33.83	0.00	0.00	0.00	0.00	0.00	0.00	**2**
56	12.91	0.00	33.13	21.70	0.00	21.57	20.24	0.00	0.00	**5**
57	26.26	23.92	35.29	0.00	0.00	0.00	0.00	0.00	0.00	**3**
58	0.00	19.79	32.21	0.00	0.00	0.00	0.00	0.00	0.00	**2**
59	17.42	16.03	28.68	0.00	0.00	0.00	0.00	0.00	0.00	**3**
60	0.00	0.00	0.00	0.00	0.00	0.00	21.36	0.00	0.00	**1**
61	0.00	0.00	32.40	0.00	0.00	0.00	31.57	0.00	35.84	**3**
62	25.06	17.73	25.68	0.00	0.00	0.00	22.61	0.00	0.00	**4**
63	20.27	13.50	24.05	0.00	0.00	0.00	0.00	0.00	0.00	**3**
64	0.00	0.00	0.00	0.00	0.00	30.62	0.00	0.00	0.00	**1**
65	30.22	28.96	0.00	0.00	0.00	0.00	34.82	0.00	0.00	**3**
66	17.70	0.00	0.00	0.00	0.00	0.00	0.00	0.00	0.00	**1**
67	10.58	22.53	26.26	0.00	0.00	0.00	21.95	0.00	0.00	**4**
68	12.97	17.09	0.00	25.47	0.00	0.00	0.00	0.00	0.00	**3**
69	21.57	16.32	0.00	0.00	0.00	0.00	0.00	0.00	0.00	**2**
70	10.41	27.10	25.05	0.00	0.00	0.00	0.00	0.00	0.00	**3**
71	0.00	28.70	0.00	0.00	0.00	0.00	0.00	0.00	0.00	**1**
72	0.00	0.00	29.37	0.00	0.00	0.00	0.00	0.00	0.00	**1**
73	27.68	0.00	0.00	0.00	0.00	0.00	21.74	0.00	0.00	**2**
74	0.00	0.00	34.43	0.00	24.89	26.27	21.75	0.00	0.00	**4**
75	16.88	0.00	0.00	28.72	24.82	26.99	26.31	0.00	29.25	**6**
76	0.00	0.00	0.00	0.00	0.00	0.00	31.07	0.00	0.00	**1**
77	26.16	16.63	30.03	0.00	0.00	0.00	0.00	0.00	0.00	**3**
78	21.40	16.44	0.00	0.00	0.00	0.00	0.00	0.00	0.00	**2**
79	0.00	24.31	0.00	0.00	0.00	0.00	0.00	0.00	0.00	**1**
80	26.39	23.82	0.00	0.00	0.00	0.00	0.00	0.00	0.00	**2**
81	0.00	19.02	30.62	0.00	0.00	0.00	0.00	0.00	0.00	**2**
82	15.06	22.78	29.92	0.00	0.00	0.00	0.00	0.00	0.00	**3**
83	14.21	0.00	0.00	0.00	0.00	0.00	0.00	0.00	26.42	**2**
84	0.00	16.52	0.00	0.00	0.00	0.00	0.00	0.00	24.67	**2**
85	15.39	22.70	0.00	0.00	0.00	0.00	21.11	0.00	0.00	**3**
86	0.00	0.00	0.00	36.85	0.00	0.00	25.31	0.00	0.00	**2**
87	0.00	33.81	0.00	0.00	0.00	0.00	0.00	0.00	0.00	**1**
88	0.00	0.00	34.07	0.00	0.00	0.00	0.00	0.00	0.00	**1**
89	26.28	22.22	0.00	0.00	0.00	0.00	0.00	0.00	0.00	**2**
90	28.03	29.40	0.00	0.00	0.00	0.00	0.00	0.00	0.00	**2**
91	0.00	27.64	0.00	0.00	0.00	0.00	0.00	0.00	0.00	**1**
92	29.50	23.52	0.00	0.00	0.00	0.00	0.00	0.00	0.00	**2**
93	0.00	28.47	0.00	0.00	0.00	0.00	0.00	0.00	0.00	**1**
94	29.61	20.49	0.00	0.00	0.00	0.00	0.00	0.00	0.00	**2**
95	0.00	24.26	0.00	0.00	0.00	0.00	0.00	0.00	0.00	**1**
96	0.00	0.00	0.00	0.00	0.00	0.00	0.00	24.68	31.09	**2**
97	0.00	0.00	0.00	0.00	0.00	0.00	0.00	30.08	29.81	**2**
98	0.00	0.00	0.00	0.00	0.00	0.00	0.00	31.02	33.77	**2**
99	0.00	0.00	36.28	0.00	0.00	0.00	0.00	28.03	0.00	**2**
100	0.00	0.00	0.00	0.00	0.00	0.00	0.00	28.38	0.00	**1**
101	0.00	0.00	0.00	0.00	0.00	0.00	0.00	27.76	31.59	**2**
102	0.00	0.00	34.43	0.00	0.00	0.00	0.00	26.75	0.00	**2**
103	0.00	0.00	0.00	0.00	0.00	0.00	0.00	23.33	0.00	**1**
104	0.00	29.28	0.00	0.00	0.00	0.00	0.00	0.00	0.00	**1**
105	0.00	27.25	0.00	0.00	0.00	0.00	0.00	0.00	0.00	**1**
106	27.26	0.00	0.00	0.00	0.00	0.00	0.00	0.00	0.00	**1**
107	0.00	0.00	0.00	0.00	0.00	0.00	0.00	0.00	35.67	**1**
108	0.00	24.52	0.00	0.00	0.00	0.00	0.00	0.00	0.00	**1**
109	0.00	25.09	0.00	0.00	0.00	0.00	0.00	0.00	0.00	**1**
110	25.34	0.00	0.00	0.00	0.00	0.00	0.00	0.00	0.00	**1**
111	30.50	0.00	0.00	0.00	0.00	0.00	0.00	0.00	0.00	**1**
112	0.00	27.79	0.00	0.00	0.00	0.00	0.00	0.00	0.00	**1**
113	26.14	0.00	0.00	0.00	0.00	0.00	0.00	0.00	0.00	**1**
114	24.57	0.00	0.00	0.00	0.00	0.00	0.00	0.00	0.00	**1**
115	33.55	0.00	0.00	0.00	0.00	0.00	0.00	0.00	0.00	**1**
116	25.84	0.00	0.00	0.00	0.00	0.00	0.00	0.00	0.00	**1**
117	28.88	0.00	0.00	0.00	0.00	0.00	0.00	0.00	0.00	**1**
118	32.00	0.00	0.00	0.00	0.00	0.00	0.00	0.00	0.00	**1**
119	29.86	0.00	0.00	0.00	0.00	0.00	0.00	0.00	0.00	**1**
120	28.96	0.00	0.00	0.00	0.00	0.00	0.00	0.00	0.00	**1**
121	28.16	0.00	0.00	0.00	0.00	0.00	0.00	0.00	0.00	**1**
122	26.21	0.00	0.00	0.00	0.00	0.00	0.00	0.00	0.00	**1**
123	27.72	0.00	0.00	0.00	0.00	0.00	0.00	0.00	0.00	**1**
124	29.13	0.00	0.00	0.00	0.00	0.00	0.00	0.00	0.00	**1**
125	26.59	0.00	0.00	0.00	0.00	0.00	0.00	0.00	0.00	**1**
126	25.45	0.00	0.00	0.00	0.00	0.00	0.00	0.00	0.00	**1**
127	28.03	0.00	0.00	0.00	0.00	0.00	0.00	0.00	0.00	**1**
128	0.00	0.00	0.00	0.00	33.04	0.00	0.00	0.00	0.00	**1**
129	27.98	0.00	0.00	0.00	0.00	0.00	0.00	0.00	0.00	**1**
130	30.75	0.00	0.00	0.00	0.00	0.00	0.00	0.00	0.00	**1**
131	0.00	25.12	0.00	0.00	32.92	0.00	0.00	0.00	0.00	**2**
132	33.77	0.00	0.00	0.00	0.00	0.00	0.00	0.00	0.00	**1**
133	27.13	0.00	0.00	0.00	0.00	0.00	0.00	0.00	0.00	**1**
134	25.28	0.00	0.00	0.00	0.00	0.00	0.00	0.00	0.00	**1**
135	31.73	0.00	0.00	0.00	0.00	0.00	0.00	0.00	0.00	**1**
136	25.68	0.00	0.00	0.00	0.00	0.00	0.00	0.00	0.00	**1**
137	27.55	0.00	0.00	0.00	0.00	0.00	0.00	0.00	0.00	**1**
138	31.14	0.00	0.00	0.00	0.00	0.00	0.00	0.00	0.00	**1**
139	29.37	0.00	0.00	0.00	0.00	0.00	0.00	0.00	0.00	**1**
NTC-1	0.00	0.00	0.00	0.00	0.00	0.00	0.00	0.00	0.00	**–**
NTC-2	0.00	0.00	0.00	0.00	0.00	0.00	0.00	0.00	0.00	**–**
NTC-3	0.00	0.00	0.00	0.00	0.00	0.00	0.00	0.00	0.00	**–**
PAC-1	26.44	26.82	26.19	26.07	27.18	26.35	26.73	29.14	28.30	**–**
PAC-2	26.69	28.37	27.94	26.12	26.42	26.22	26.47	28.69	26.34	**–**
PAC-3	26.59	28.14	27.52	25.73	26.21	26.14	26.29	28.25	26.21	**–**
**No. of positive**	**73**	**57**	**22**	**6**	**6**	**11**	**35**	**8**	**13**	

### Canine clinical sample collection and nucleic acid extraction

Detailed information for clinical sample collection and nucleic acid extraction were provided in Dong et al., 2022 [4]. As described, a total of 740 canine clinical samples were used for diagnostic validation of the new assay. Nucleic acids were extracted by MagMax™ Viral/Pathogen Nucleic Acid Isolation Kit (Applied Biosystems/ThermoFisher, Foster City, CA, USA) according to the manufacturer's recommendations. Extracted nucleic acids were tested with the newly developed CIRD panel RT-PCR assay, and compared with an older version of the panel test that detects the same pathogens, except that it does not differentiate *M. cynos* from *M. canis*.

### Real-time PCR conditions

Detailed PCR reaction composition and thermocycling profile were provided in Dong et al., 2022 [4]. To streamline the reaction preparations, all real-time PCR and RT-PCR reactions, including those for RNA and DNA targets, were performed using Path-ID™ One-Step RT PCR Kit (Applied Biosystems, Thermo Fisher, Hillsboro, OR, USA) with Bio-Rad CFX96™ Touch™ Real-time PCR Detection System. Real-time PCR reactions were prepared in a 20 µL of reaction that was composed of 0.5 µM of each forward and reverse primer, and 0.15 µM of each probe, 5 µL of template, 10 µL of Multiplex RT-PCR buffer, and 2 µL of Multiplex Enzyme Mix. The reaction parameters contained: reverse transcription at 48 °C for 10 mins, inactivation and initial denaturation at 95 °C for 10 mins, followed by 45 cycles of 95 °C for 15 s, and 60 °C for 45 s. The threshold cycle (Ct) values were analyzed and generated by Bio-Rad CFX Manager 3.0 software.

### Sequencing confirmation

Sequencing primer information is listed in Table 2 of Dong et al., 2022 [4]. Briefly, primers amplifying a fragment from each pathogen that encompassing the RT-qPCR primer binding region were designed and used for Sanger sequencing confirmation of selected positive samples.

**Table 2:** Effect of three different concentrations of canine distemper virus on detection of canine influenza virus and canine respiratory coronavirus in Subpanel 3 of the assay.

	CDV dilutions	CIV Ct	Delta Ct	CRCoV	Delta Ct
CIV and CRCoV positive, #1	No CDV (CK)	27.31		31.25	
	CDV 1:1	28.77	*1.47*	32.02	*0.77*
	CDV 1:100	28.63	*1.33*	32.22	*0.97*
	CDV 1:10,000	28.68	*1.38*	32.1	*0.85*
Standard deviation (SD)		0.69		0.44	
Coefficient of variation (CV)		2.45 %		1.38 %	
CIV and CRCoV positive, #2	No CDV	31.41		28.14	
	CDV 1:1	33.23	*1.82*	28.76	*0.62*
	CDV 1:100	33.12	*1.7*	28.61	*0.47*
	CDV 1:10,000	32.7	*1.29*	29.3	*1.16*
Standard deviation (SD)		0.84		0.48	
Coefficient of variation (CV)		2.56 %		1.67 %	
CIV positive, CRCoV negative	No CDV	28.08		0	
	CDV 1:1	29.66	*1.58*	0	*–*
	CDV 1:100	29.58	*1.5*	0	*–*
	CDV 1:10,000	29.75	*1.67*	0	*–*
Standard deviation (SD)		0.79			
Coefficient of variation (CV)		2.72 %			
CIV negative, CRCoV positive	No CDV	0	–	33.84	*–*
	CDV 1:1	0	–	35.35	*1.51*
	CDV 1:100	0	–	35.49	*1.65*
	CDV 1:10,000	0	–	34.86	*1.02*
Standard deviation (SD)				0.75	
Coefficient of variation (CV)				2.14 %	

### Preparation of positive control plasmids

For quantification purposes, a fragment encompassing the RT-qPCR primer binding region for eight of the nine pathogen targets was amplified from clinical samples using the sequencing primers listed in Table 2 of Dong et al., 2022 [4]. Amplified fragments were cloned and used as positive amplification controls. We did not have a *B. brochiseptica* positive sample in the beginning of the project, and the target was synthesized and cloned. Insert sequences of all nine plasmids were confirmed by Sanger sequencing. Plasmids were extracted using the Qiagen QIAamp plasmid mini kit. Plasmid DNA concentration was measured using a Nanodrop (ThermoFisher) spectrophotometer. RNA templates for the four RNA viruses, CPIV, CDV, CIA, and CRCoV, were also synthesized using MEGAscript T7 Transcription Kit, and purified with MEGAclear Kit (Invitrogen/ThermoFisher, Waltham, MA).

### Analytical sensitivity

Cloned plasmids of each pathogen were used to generate standard curves in singular and multiplex real-time PCR reactions. RNA was also synthesized through *in vitro* transcription using positive control plasmids for RNA viruses, and compared to standard curves using plasmid DNA. The copy number of each target gene was calculated using the following formula [17,18]:

$$\text{Plasmid}\;\text{DNA}\;\text{copies}/\mu L=\frac{\mu L=\left(6.02\times 10^{23}\right)\times \left(\mathrm{Xng}/\mu L\times 10^{-9}\right)}{\text{Plasmid}\;\text{length}\;\left(\text{bp}\right)\times 660}$$

$$\text{RNA}\;\text{copies}/\mu L=\frac{\left(6.02\times 10^{23}\right)\times \left(\mathrm{Xng}/\mu L\times 10^{-9}\right)}{\text{RNA}\;\text{length}\;\left(\text{bp}\right)\times 340}$$

X: Concentration in *ng/µL* measured by Nanodrop spectrophotometer.

### Analytical specificity

Selected positive samples for each targeted pathogen, especially those had discrepant results between the old and new assays, were sequenced using primers listed in Dong et al., 2022 [4]. Specificity was also tested with nine specific-target negative pools of the control plasmids, from which each pool contained all but one of the target plasmids, to test if any signals can be detected for the missing target in each pool [4].

### Statistical analysis

Standard deviation and coefficient of variation were applied to data sets presented in Tables 2 and 3. Standard deviation was calculated using the “STDEV” function in Microsoft Excel 2019; coefficient of variation was calculated by dividing the standard deviation with the mean of the given set of data.

**Table 3:** Comparison of lung tissue and nasal swab in detecting the nine CIRD pathogens by spiking DNA and/or RNA templates to negative lung or nasal samples.

	Sample	Subpanel 1			Subpanel 2			Subpanel 3			
		M. cynos	M. canis	B. br	CAV2	CHV1	CPIV	CDV	CIV	CrCoV	cGAPDH
DNA	Lung 1	19.26	19.7	21.16	17.48	16.76	18.36	21.72	20.58	22.16	20.23
	Swab 1	19.46	19.91	23.73	18.04	17.36	18.9	21.74	20.54	22.34	25.79
	SD*	0.14	0.15	1.82	0.40	0.42	0.38	0.01	0.03	0.13	
	CV*	0.73 %	0.75 %	8.10 %	2.23 %	2.49 %	2.05 %	0.07 %	0.14 %	0.57 %	
	Lung 2	26.34	26.91	28.4	24.5	23.8	25.3	30.23	29.91	30.61	20.34
	Swab 2	27.08	28.1	29.11	24.78	24.12	25.57	28.98	28.44	29.32	25.25
	SD	0.52	0.84	0.50	0.20	0.23	0.19	0.88	1.04	0.91	
	CV	1.96 %	3.06 %	1.75 %	0.80 %	0.94 %	0.75 %	2.99 %	3.56 %	3.04 %	
	Lung 3	33.23	33.14	35.87	30.9	30.43	31.62	35.16	38.51	37.21	20.63
	Swab 3	32.79	33.18	36.64	31.35	30.95	32.24	34.42	35.26	36.65	25.25
	SD	0.31	0.03	0.54	0.32	0.37	0.44	0.52	2.30	0.40	
	CV	0.94 %	0.09 %	1.50 %	1.02 %	1.20 %	1.37 %	1.50 %	6.23 %	1.07 %	
RNA	Lung 1	N/A	N/A	N/A	N/A	N/A	21.14	21.16	18.24	18.6	21.08
	Swab 1	N/A	N/A	N/A	N/A	N/A	20.82	21.16	18.18	17.95	29.08
	SD	N/A	N/A	N/A	N/A	N/A	0.23	0.00	0.04	0.46	
	CV	N/A	N/A	N/A	N/A	N/A	1.08 %	0.00 %	0.23 %	2.52 %	
	Lung 2	N/A	N/A	N/A	N/A	N/A	27.02	27.45	25.34	23.98	21.01
	Swab 2	N/A	N/A	N/A	N/A	N/A	27.1	27.85	25.16	24.46	27.47
	SD	N/A	N/A	N/A	N/A	N/A	0.06	0.28	0.13	0.34	
	CV	N/A	N/A	N/A	N/A	N/A	0.21 %	1.02 %	0.50 %	1.40 %	
	Lung 3	N/A	N/A	N/A	N/A	N/A	33.74	35.11	32.44	30.11	20.93
	Swab 3	N/A	N/A	N/A	N/A	N/A	33.83	37.59	32.64	30.63	28
	SD	N/A	N/A	N/A	N/A	N/A	0.06	1.75	0.14	0.37	
	CV	N/A	N/A	N/A	N/A	N/A	0.19 %	4.82 %	0.43 %	1.21 %	

⁎ SD: Standard deviation; CV: Coefficient of variation.

### Method validation

A collection of 740 canine respiratory samples submitted by clients between 2019 and 2021 were used to evaluate a newly developed 3-panel, 9-pathogen RT-PCR assay [4]. From the 740 canine respiratory diagnostic samples, 139 were identified positive to at least one of the nine pathogens. The most prevalent pathogen detected was the pathogenic *Mycoplasma* species, *M. cynos,* which was positive in 73 samples; the next prevalent pathogen was the less pathogenic *Mycoplasma* species, *M. canis* that was positive in 57 samples, followed by 35 CDV positives; 22 *B bronchiseptica* positives; 13 CRCoV positives; 11 CPIV positives; 8 CIV positives; 6 CAdV-2 positives; and 6 CHV-1 positives. Apparently, co-infection may have occurred among these samples. Twenty-seven *M. cynos* positive samples were also positive to *M. canis*; 24 of the CDV positives were positive to one or more other pathogens; 14 *B bronchiseptica* positive samples were positive to one or both *Mycoplasma* species, and five more were positive to one or more of the viral pathogens. The sample with the most pathogens detected was sample # 75, which was positive to six of the nine pathogens, and all with relatively low threshold cycle (Ct) values: *M. cynos* (Ct=16.9), CadV-2 (Ct=28.7), CHV-1 (Ct=24.8), CPIV (Ct=27.0), CDV (Ct=26.3) and CRCoV (Ct=29.3). Additionally, two other samples were positive to five pathogens, four more samples were positive to four pathogens, 14 were positive to three pathogens, and 39 were positive to two pathogens. Collectively, 60 (43.2 %) of the 139 samples were positive to more than one pathogens (Table 1). The Ct values of all positive samples ranged from 10.4 to 36.9, with an average Ct value of 25.9, when the positive Ct cutoff value was set to 37. Raw Ct values of these 139 positive samples for each pathogen, number of positive targets in each sample, and total number of positive samples for each pathogen is summarized in Table 1.

To evaluate if different concentrations, especially a high concentration, of a pathogen are affecting the detection of other pathogens in the same reaction, we have identified a CDV positive diagnostic sample with relatively low Ct value (Ct=19), and spiked this sample with 3 different concentrations (original plus two 100-fold dilutions) into two CIV and CRCoV positive, one CIV positive but CRCoV negative, and one CIV negative and CRCoV positive diagnostic samples, and tested with Subpanel 3 of the PCR panel assay. The results indicated there was slightly increase in Cts for CIA and CRCoV targets (0.47–1.82 Cts, Table 2). Standard deviations and coefficients of variation were between 0.44–0.84 and 1.38 %−2.72 %, respectively, indicating there was no significant effect of high-concentration target on the detection of low-concentration ones. Practically, the slightly increased Ct values for ICV and CRCoV may not affect the interpretation of diagnostic results, as the variations are among those found in routine diagnostic replications, and the pathogens with lower Cts are more likely the causative agents than those with higher Ct values.

Nasal swab was the most common sample submitted for diagnosis, along with certain number of lung tissues. To illustrate if these two common types of canine respiratory samples will affect detection sensitivity, three different concentrations of DNA (plasmids) or RNA (*in vitro* transcribed) were spiked into all-target negative nasal or lung samples. Nucleic acids were extracted and tested with this panel PCR assay. Results indicated that only minor differences between the two sample types were observed with relatively low standard deviation and coefficient of variation (Table 3). As the minor differences between lung tissue and nasal swab are among the range of typical variations of technical replications in real-time PCR, they may not affect diagnostic interpretations.

## CRediT authorship contribution statement

**Junsheng Dong:** Data curation, Writing – original draft. **Wai Ning Tiffany Tsui:** Data curation, Writing – original draft. **Xue Leng:** Data curation, Investigation. **Jinping Fu:** Data curation, Investigation. **Molly Lohman:** Data curation, Investigation. **Joseph Anderson:** Data curation, Investigation. **Vaughn Hamill:** Data curation, Investigation. **Nanyan Lu:** Data curation. **Elizabeth Poulsen Porter:** Data curation, Investigation. **Mark Gray:** Data curation, Investigation. **Tesfaalem Sebhatu:** Data curation, Investigation. **Susan Brown:** Data curation. **Roman Pogranichniy:** Project administration, Writing – review & editing, Conceptualization. **Heng Wang:** Project administration, Writing – review & editing, Conceptualization. **Lance Noll:** Project administration, Writing – review & editing, Conceptualization. **Jianfa Bai:** Project administration, Writing – review & editing, Conceptualization.

## Declaration of Competing Interest

The authors declare that they have no known competing financial interests or personal relationships that could have appeared to influence the work reported in this paper.

## Data Availability

No data was used for the research described in the article.
